# Changes in Cell Wall Properties Coincide with Overexpression of Extensin Fusion Proteins in Suspension Cultured Tobacco Cells

**DOI:** 10.1371/journal.pone.0115906

**Published:** 2014-12-23

**Authors:** Li Tan, Yunqiao Pu, Sivakumar Pattathil, Utku Avci, Jin Qian, Allison Arter, Liwei Chen, Michael G. Hahn, Arthur J. Ragauskas, Marcia J. Kieliszewski

**Affiliations:** 1 Complex Carbohydrate Research Center, University of Georgia, Athens, Georgia, United States of America; 2 BioEnergy Science Center, University of Georgia, Athens, Georgia, United States of America; 3 Department of Plant Biology, University of Georgia, Athens, Georgia, United States of America; 4 Biosciences Division, Oak Ridge National Laboratory, Oak Ridge, Tennessee, United States of America; 5 Department of Chemistry and Biochemistry, Ohio University, Athens, Ohio, United States of America; 6 Suzhou Institute of Nano-Tech and Nano-Bionics (SINANO), Suzhou, China; The University of Melbourne, Australia

## Abstract

Extensins are one subfamily of the cell wall hydroxyproline-rich glycoproteins, containing characteristic SerHyp_4_ glycosylation motifs and intermolecular cross-linking motifs such as the TyrXaaTyr sequence. Extensins are believed to form a cross-linked network in the plant cell wall through the tyrosine-derivatives isodityrosine, pulcherosine, and di-isodityrosine. Overexpression of three synthetic genes encoding different elastin-arabinogalactan protein-extensin hybrids in tobacco suspension cultured cells yielded novel cross-linking glycoproteins that shared features of the extensins, arabinogalactan proteins and elastin. The cell wall properties of the three transgenic cell lines were all changed, but in different ways. One transgenic cell line showed decreased cellulose crystallinity and increased wall xyloglucan content; the second transgenic cell line contained dramatically increased hydration capacity and notably increased cell wall biomass, increased di-isodityrosine, and increased protein content; the third transgenic cell line displayed wall phenotypes similar to wild type cells, except changed xyloglucan epitope extractability. These data indicate that overexpression of modified extensins may be a route to engineer plants for bioenergy and biomaterial production.

## Introduction

Plants are the major source of food and chemicals on earth. With our intensifying desire for sustainable energy, the engineering of plants, in particular their cell walls, to achieve greater biofuel production is a major focus of current cell wall research. Genetic manipulation of plants using gene transformation or selective breeding allows the production plants possessing higher biomass and digestibility [1]–[3].

One approach is to modify the enzymes involved in wall polymer biosynthesis. Examples include the modification of caffeic acid O-methyltransferase (*COMT*) and caffeoyl CoA 3-O-methyltransferase (*CCoAOMT*) [4], [5], two enzymes within the lignin biosynthesis pathway, to reduce lignin content and/or modify its structure for higher saccharification of biomass. Down-regulation of the *COMT* gene in switchgrass made the wall more amenable to degradation [4]. The modification resulted in low lignin, a low syringyl:guaiacyl lignin ratio in the transgenic plants, and a 8% increase in ethanol production during traditional fermentation. Similarly, suppression of *CCoAOMT* in transgenic poplar tree reduced cell wall lignin content as much as 10% and significantly increased glucose yield from mature poplar wood when subjected to enzymatic digestion [5].

To reduce the costs associated with chemical pretreatment of biomass, another approach involves expression of wall glycan degrading enzymes such as cellulases and hemicellulases *in planta*. Examples of this approach include expression of *Clostridium thermocellum* xylanase (xynZ) in the apoplast of transgenic tobacco [6] and expression of *Acidothermus celulolyticus* endoglucanase E1 in tobacco chloroplasts [7]. Interestingly, E1 protein was biologically active in both fresh and dried leaves. Expression of a poplar cellulase (*PaPopCel1*) in *Arabidopsis* resulted in cell elongation and subsequent increased cell size, most likely because the poplar cellulase removed disordered glucose from the cellulose microfibrils, which probably reduced their cross-links with xyloglucans [8]. Transgenic maize expressing xylanase XynA, or endoglucanase, or both enzymes, yielded up to 141% higher glucose and 172% higher xylose compared to control plants. This resulted in a total of 55% increase in ethanol production [9].

Here we report an approach to changing the cell wall properties by overexpressing cross-linkable chimeric P3 extensin analogs that also contain repeats of an AGP glycosylation-motif and repeats of the human elastin peptide VPGVG in tobacco (*Nicotiana tabacum*) BY-2 (Bright Yellow) suspension cultured cells. Extensins belong to a superfamily of plant cell wall glycoproteins, the hydroxyproline-rich glycoproteins (HRGPs), which also include proline-rich proteins (PRPs) and arabinogalactan-proteins (AGPs) [10]. Unlike the AGPs that contain clustered AlaHyp, SerHyp, and ThrHyp glycosylation-motifs and variations thereof, allowing for arabinogalactan polysaccharide addition [11], [12], extensins share a diagnostic repetitive SerHyp_4_ glycosylation-motif that directs Hyp-O-glycosylation with short oligoarabinosides [13]. Extensins often include Tyr-containing cross-linking motifs, such as ValTyrLys in P1-type extensins and TyrXTyrLys (X represents any amino acid except Pro) in P3-type extensins [10]. These motifs lead to the formation of the cross-linked tyrosine derivatives, including isodityrosine (Idt), pulcherosine, and di-Idt. Pulcherosine and di-Idt covalently cross-link extensin monomers to form protein scaffolds in the cell wall [14]–[17]. The cross-linking property of extensins provides a potential route to modify the plant cell wall.

Elastin is a cross-linked protein distributed throughout the animal extracellular matrix, such as arteries, lungs, elastic ligaments, bladder, and skin [18], [19], imparting elasticity to those tissues. Due to the number of cross-links formed through Lys residues, elastin is highly insoluble. However, its precursor, tropoelastin, is soluble and composed of two major domains, a hydrophilic cross-linkable domain rich in Lys and Ala residues, and a hydrophobic elastic domain rich in Val, Pro, Gly, and Ala residues. The elastic domain contains repetitive amino acid motifs, including the peptides VPGG, VPGVG, APGVGV, and VPGFGVGAG [20]. Among these sequences, the pentapeptide VPGVG is the most common element with more than 50 such repeats occurring in one elastin molecule. We aimed to change cell wall properties by introducing the exogenous elastin motifs, as well as AGP motifs, into the wall through the synthetic extensin analogs [17].

The cell wall properties of three different transgenic cell lines were all changed, but in different ways. One transgenic cell line was observed with a decreased cellulose crystallinity and increased wall xyloglucan content; the second transgenic cell line contained dramatically increased hydration capacity and notably increased cell wall biomass, increased di-isodityrosine, and increased protein content; the third transgenic cell line displayed wall phenotypes similar to wild type cells, except changed extractability of xyloglucan epitopes. These results suggest that wall extensin modification has potential as an approach to manipulate wall structure, in some instances resulting in greater sugar release for bioenergy production.

## Materials and Methods

### 1. Construction of binary vectors

**Figure 1:**
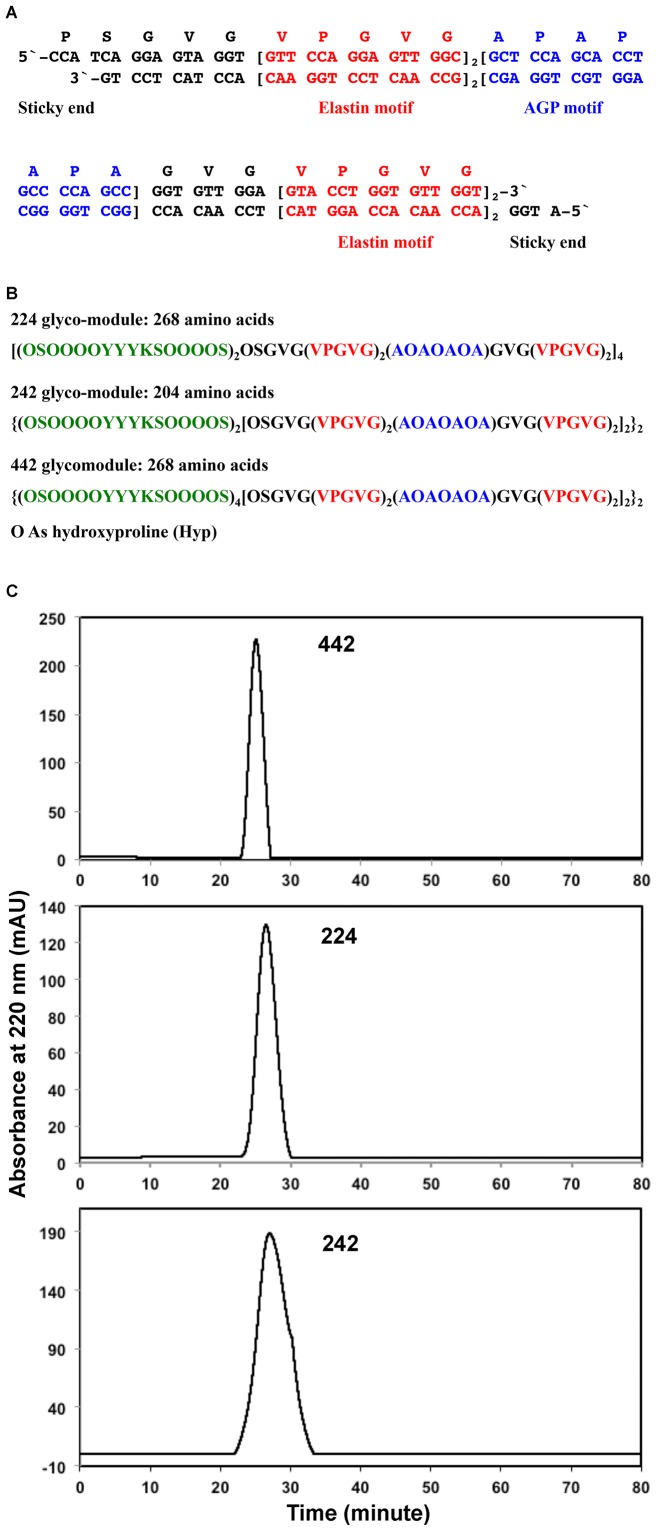
Designed nucleotides (A), deduced protein sequences (B), and purification of 442 glycomodule (C). A. Designed nucleotides encode four repeats of human elastin motifs separated by an AGP motif Ala-Pro-Ala-Pro-Ala-Pro-Ala, with two sticky ends for cloning. B. Predicted polypeptide sequence of each glycomodule based on previous work on synthetic gene products. However, based on amino acid composition analysis, Pro residues in the elastin motifs were also hydroxylated. C. Size exclusion chromatograms of the three glycomodules. Each glycomodule was loaded and eluted on a Superose-12 analytical size exclusion column. The retention time of 242 was 28 min, while those of 224 and 442 were 26 min, which is consistent with the calculated molecular weights for 242 (55.9 kDa), 224 (68.7 kDa) and 442 (68.7 kDa).

Two oligonucleotides, 5′-CCATCAGGAGTAGGTGTTCCAGGAGTTGGCGTTCC-AGGAGTTGGCGCTCCAGCACCTGCCCCAGCCGGTGTTGGAGTACCTGGTGTTGGTGTACCTGGTGTTGGT-3′ and 5′-TACCTGGTTGTGGTCCATGTGGTTGTGG-TCCATGAGGTTGTGGCCGACCCCGACCACGACCTCGCGGTTGAGGACCTTGCGGTTGAGGACCTTGTGGATGAGGAC-3′, were designed encoding an AGP motif (Ala-Pro-Ala-Pro-Ala-Pro) flanked by two repeats of human elastin motif Val-Pro-Gly-Val-Gly at each end (Fig. 1A). The oligonucleotide set was abbreviated as *E_2_AE_2_* (E: elastin motif; A: AGP motif).

Fifty nanograms of each above oligonucleotide were annealed in 1X ligase buffer and ligated to a *pUC18* vector between the BbsI and BsmF1 restriction sites. The resulting plasmid was named as *pUC-E_2_AE_2_*, which was digested by two sets of restriction enzymes BbsI/ScaI and BsmFI/ScaI, respectively [17]. The 1.1 kb BsmFI-ScaI fragment and the 1.8 kb BbsI-ScaI fragments were ligated to form a vector containing two repeats of *E_2_AE_2_*. The corresponding plasmid was named *pUC-E_2_AE_4_AE_2_*.

By using the same strategy shown above, the BsmFI-ScaI fragment of *pUC-YK_2_* or *pUC-YK_n_* [17] containing the *YK_2_* or *YK_4_* gene was ligated with the BbsI-ScaI fragment of *pUC-E_2_AE_2_* and *pUC-E_2_AE_4_AE_2_*, respectively, which lead to the formation of *pUC-YK_2_-E_2_AE_2_* and *pUC-YK_2_-E_2_AE_4_AE_2_*, and *pUC-YK_4_-E_2_AE_4_AE_2_*. Here, *YK* stands for gene encoding peptide Ser-Pro_4_-Ser-Pro-Ser-Pro_4_-Tyr-Tyr-Tyr-Lys [17].

Similarly, *pUC-YK_2_-E_2_AE_2_*, *pUC-YK_2_-E_2_AE_4_AE_2_*, and *pUC-YK_4_-E_2_AE_4_AE_2_* were dimerized with themselves, respectively. The desired gene sizes of *(YK_2_-E_2_AE_4_AE_2_)_2_* and *(YK_4_-E_2_AE_4_AE_2_)_2_* were about 600 and 800 bps. The corresponding plasmids were named *pUC-(YK_2_-E_2_AE_4_AE_2_)_2_* and *pUC-(YK_4_-E_2_AE_4_AE_2_)_2_*. The obtained *pUC-(YK_2_-E_2_AE_2_)_2_* was further dimerized, formed *pUC-(YK_2_-E_2_AE_2_)_4_* with a gene size about 800 bps. From now on, the *(YK_2_-E_2_AE_4_AE_2_)_2_*, *(YK_4_-E_2_AE_4_AE_2_)_2_*, and *(YK_2_-E_2_AE_2_)_4_* genes were abbreviated as *242*, *442*, and *224*, respectively.

The XmaI-NcoI fragments containing the synthetic genes in *pUC-224*, *pUC-242*, and *pUC-442* were ligated into *pUC-SS^tob^-(AP)_51_-EGFP*, respectively, to replace the *(AP)_51_* fragment (*(AP)_51_* stands for a synthetic gene encoding 51 repeats of Ala-Pro [12], and *SS^tob^* stands for the signal sequence of a tobacco extensin [11]). The three new plasmids were named as *pUC-SS^tob^-224-EGFP*, *pUC-SS^tob^-242-EGFP,* and *pUC-SS^tob^-442-EGFP*. The gene cassettes were further cloned into a *pBI121* vector between the BamHI and SacI restriction sites, formed plant transformation vectors *pBI121-SS^tob^-224-EGFP*, *pBI121-SS^tob^-242-EGFP*, and *pBI121-SS^tob^-442-EGFP*. All gene sequences were confirmed by DNA sequencing.

### 2. Transfer of the genes into tobacco cells

One hundred nanograms of above each constructed *pBI121* plasmid were transformed into *Agrobacterium tumefaciens* strain LBA4404 by the freeze-thaw method [21]. Positive colonies of *Agrobacterium* were selected via Kanamycin/Streptomycin resistance. The transformed *Agrobacteria* were used to co-culture with 4-day-old tobacco BY2 cells at 28°C for 2 days [12]. The infected tobacco cells were washed 4 times with Schenk and Hildebrandt (SH) culture media and were spread on SH solid plates with Kanamycin (100 µg/ml) and Timentin (200 µg/ml). The Kanamycin-selected cells were subcultured in SH culture media as described earlier [12].

### 3. Purification of fusion proteins and glycomodules from suspension culture media

The culture media of 20-day-old transgenic tobacco BY2 cells were filtered from the cultures, concentrated through rotary evaporation, and dialyzed against distilled deionized (d.d.) H_2_O. The EGFP fusion proteins in the dialyzed medium were separated via a combination of chromatography, including hydrophobic interaction (HIC) (Phenyl Sepharose 6 Fast Flow, 16×700 mm, Amersham-Pharmacia Biotech) and C-18 reverse-phase (10 µm, PRP-1, 7×305 mm, Hamilton) as described [12].

Eight to ten milligrams of 224-EGFP, 242-EGFP, and 442-EGFP were dissolved in 500 µl of d.d. H_2_O and denatured at 100°C for 5 min. Five hundred µl of 4% (w/v) ammonium bicarbonate and 10 µl of 10 µg/µl trypsin were added to the solution, which were incubated at room temperature for 1 day. The resulted 224, 242, and 442 glycomodules were purified from the digestion mixture, respectively, through a semipreparative Superose-12 gel filtration (16×500 mm, Amersham-Pharmacia Biotech) using 50 mM sodium phosphate buffer (pH 7.0) and C-18 reverse-phase (5 µm, PRP-1, 4.1×150 mm, Hamilton) chromatography as reported [12].

### 4. Cell wall preparations of suspension cultured cells

Forty grams of above filtered fresh cells from each suspension culture were re-suspended in 80 ml of suspension buffer (Tris-HCl, 50 mM, pH 7.0, 0.1% BSA (w/v), 1% sucrose, 0.1% sodium metabisulfite) and cooled on ice, followed by sonication for 12 minutes on ice (30 second sonication and 1 minute interval cycles). The cell debris was spin at 3000×g for 5 minutes. The supernatant was discarded. The cell walls were washed with 600 ml of 1 M NaCl and then with 2 L of d.d. H_2_O until the conductivity of the supernatant equal to that of d.d. H_2_O. The resulted cell walls were lyophilized.

Cell walls used for glycosyl composition, IDT and di-IDT measurement, glycome profiling, sugar release, and solid state NMR analyses were prepared as follows [22]: Forty grams of filtered cells were washed with 100 mM potassium phosphate (pH 7.0) and 500 mM potassium phosphate (pH 7.0), broken in 500 mM potassium phosphate (pH 7.0). The cell walls were washed in order with 500 mM potassium phosphate (pH 7.0), d.d. H_2_O, CHCl_3_/MeOH (1∶1), and acetone, and air-dried in a hood.

### 5. Coprecipitation with (β-D-Galactosyl)_3_-Yariv reagent

Three hundred µl of each sample (100 µg/300 µl distilled water) were mixed with 300 µl of (β-D-Galactosyl)_3_-Yariv reagent (1 mg/ml in 2% [w/v] NaCl aqueous solution). The mixture was incubated at room temperature for 1 hour followed by pelleting the precipitate in a microfuge. The pellets were washed with 2% NaCl solution, then redissolved in 0.1 M NaOH. The absorbance of the resulting solution was measured at 420 nm [12]. The characterized glycomodule (AlaHyp)_51_ was used as a standard AGP [12].

### 6. Glycosyl composition analysis of purified glycomodules and cell wall preparations

For neutral sugar analysis, 60 µg of each glycomodule or 100 µg of each cell wall preparation were hydrolyzed in 200 µl of 2N TFA at 121°C for 1 hr. The neutral monosaccharides were analyzed as alditol acetate derivatives by gas chromatography as described [12]. Uronic acids were quantified by colorimetric method using m-hydroxydiphenyl [23].

### 7. Amino acid composition and Hyp content analysis of purified glycomodules and cell walls

The amino acid compositions of glycomodules were analyzed as reported [24]. Hyp contents in cell walls were measured as described earlier [25].

### 8. Tyr, IDT, and di-IDT measurement of cell wall preparations

The Tyr, IDT, and di-IDT amounts of each cell wall preparation were analyzed as described earlier [17]. One hundred µg of each cell wall preparation were hydrolyzed in 200 µl of 6 N HCl with 10 mM phenol at 110°C for 20 hr. Hydrolysates were dried under nitrogen gas. The residues were separated on a polyhydroxyethyl A (10 nm, 9.4×200 mm, Poly LC Inc) gel filtration column [17]. The IDT and di-IDT in each cell wall preparation were quantified by comparing with IDT and di-IDT standards (gift from professor Stephen Fry, University of Edinburgh).

### 9. In vitro cross-linking of EGFP fusion proteins and glycomodules

*In vitro* cross-linking reactions of the glycomodules or fusion glycoproteins were carried out using the tomato pI 4.6 extensin peroxidase fraction isolated from tomato cell suspension cultures as reported earlier [17], [26].

### 10. Confocal microscopy study

Suspension cultured cells were checked for green fluorescence under a Zeiss LSM 510 laser-scanning confocal microscope, with excitation wavelength at 488 nm and emission wavelength at 510 nm. The cells were inspected either in SH culture media or in 1 M mannitol for plasmolysis.

### 11. Glycome profiling of cell wall preparations

Extraction of cell walls with 50 mM ammonium oxalate, 50 mM sodium carbonate, 1 M KOH, 4 M KOH, sodium chlorite in acetic acid, and post chlorite 4 M KOH were carried out following the method described earlier [27], [28]. Glycome profiling of wall glycans in the extracts using ELISA analysis to monitor the glycan epitopes were carried out as reported [27], [28]. ELISA assays were done on an equal sugar basis. All ELISA wells were coated with 15 µl of a stock solution containing 20 µg/ml carbohydrate (glucose equivalents).

### 12. Analysis of glucose release from cell wall preparations

Cellulase from *Trichoderma reesei* ATCC 26921 and Novozyme 188 from *Aspergillus niger* were used. Enzymatic hydrolysis of cell walls was performed on a 1% (w/v) suspension in 50 mM citrate buffer (pH 4.8) with cellulase and β-glucosidase loadings of 20 FPU/g and 40 CBU/g, respectively. The mixture was incubated at 50°C under continuous agitation at 150 rpm with antibiotic antimycotic solution (1%, v/v) added. Samples of hydrolysis liquid (0.5 mL) at time intervals of 1, 2, 4, 12, 24, and 48 hr were withdrawn and the hydrolysis was quenched by submersion for 10 min in a vigorously boiling water bath. The liquid samples were then immediately frozen at −20°C until analysis on an Agilent 1200 series HPLC system (Agilent Technologies) equipped with an autosampler and an Aminex HPX-87H column and pre-column (Bio-rad Laboratories). The analysis was carried out at 65°C using 10 mM nitric acid as eluent at a flow rate of 0.6 mL min^−1^ and with refractive index detection. The calibration of the system was performed with glucose standards.

### 13. Solid state NMR analysis

The cell wall samples were characterized by solid state cross polarization/magic angle spinning (CP/MAS) ^13^C nuclear magnetic resonance (NMR) using a Bruker Avance III-400 spectrometer operating at 100.59 MHz for ^13^C nuclei as described [29]. The sample (∼60% moisture) was packed in a 4 mm cylindrical ceramic MAS rotor and spun at 10 kHz. The CP/MAS NMR spectra were acquired using a 5 µs (90°) proton pulse, 1.5 ms contact time, 4.0-s recycle delay and 8–12 k scans. Bruker's TopSpin 2.1 software and NUTS NMR software (Acorn NMR, Inc., Livermore, CA) were used for processing the NMR spectral data.

### 14. Measurement of the radius of gyration of glycomodules

The effect of temperature on glycomodule conformations was measured using a DynaPro Titan and ProteinSolutions Temperature controlled MicroSmapler (Wyatt Technology, Santa Barbara, CA). Five hundred µg of each glycomodule were dissolved in 1 ml of d.d. water. Radii of gyration were measured at 5°C, 8°C, 10°C, 14°C, 20°C, and 24°C. Five readings were taken at each temperature.

## Results

### 1. Each transgenic tobacco cell line produced the corresponding fusion glycoprotein

The *SS^tob^-224-EGFP*, *SS^tob^-242-EGFP*, and *SS^tob^-442-EGFP* genes cassettes were cloned into a *pBI121* binary vector and transformed into suspension cultured tobacco BY2 cells (Fig. 1A and 1B). After selection via kanamycin resistance, at least two cell lines of each construct were cultured independently in liquid culture media. The green fluorescent fusion glycoproteins were purified from the culture media as described earlier [12]. The corresponding fusion glycoproteins 224-EGFP, 242-EGFP, and 442-EGFP were treated with trypsin to cleave the EGFP tag. The resulting 224, 242, and 442 hybrid glycomodules (Fig. 1B) were further purified via size exclusion (SEC) and reversed phase chromatography as previously reported [12], [17]. The retention times of the glycomodules on SEC were consistent with the estimated molecular weights of 55.9 kDa, 68.7 kDa, and 68.7 kDa for 242, 224, and 442, respectively, calculated based on the masses of the polypeptides and the attached glycans (Fig. 1C) [12], [17].

Amino acid composition analyses revealed that the mole percent of each amino acid in each glycomodule was close to the predicted composition, except for the higher content of Hyp and lower amount of Pro (Table 1). This may have resulted from the hydroxylation of Pro residues in the elastin motifs (Fig. 1B). The amino acid compositions indicated the glycomodules were the expected gene products and were consistent with earlier results obtained from expression of other extensin and AGP analogs in tobacco cells [11]–[13], [17].

**Table 1:** Amino acid composition of three glycomodules isolated from the culture media of individual transgenic cell line.

Amino acids	224		242		442	
	Measured	Putative	Measured	Putative	Measured	Putative
O	37.0	32.8	30.1	25.5	36.4	32.8
S	11.6	10.4	8.9	7.8	11.8	10.4
G	18.2	17.9	23.6	23.5	18.6	17.9
A	8.1	6.0	9.9	7.8	8.5	6.0
P	1.6	6.0	2.6	7.8	2.3	6.0
Y	4.8	9.0	3.8	5.9	3.6	9.0
V	15.8	14.9	19.6	19.6	15.9	14.9
K	2.9	3.0	1.6	2.0	2.8	3.0

The data were shown as molar percentage and were average of two data sets.

Amino acid residues in total weight percentage (w/w): 224, 13.5%; 242, 11.6%; 442, 11.7%. The low Y contents were due to the easy degradation of Y residues during hydrolysis.

The 224, 242, and 442 glycomodules reacted with β-Gal Yariv regent, the dissolved precipitate giving the following absorbances at 420-nm: 0.432, 0.302, and 0.210, respectively, compared to 0.305 of a synthetic AGP glycomodule, (AlaHyp)_51_ [12]. This indicated that the AGP motifs in 224, 242 and 442 were glycosylated with type II arabinogalactans (AG). Glycosyl composition analyses demonstrated that 224, 242 and 442 all contained mainly arabinose, with lesser amounts of Gal, Rha, and uronic acid residues (Table 2). These results were consistent with the Hyp-contiguity hypothesis, which predicts the AG addition to the Hyp residues in the AlaHypAlaHypAlaHypAla motifs and attachment of oligoarabinosides to Hyp residues in the SerHyp_4_ extensin motifs [30].

**Table 2:** Sugar composition of three glycomodules isolated from the culture media of individual transgenic cell line.

Sugar residues	224	242	442
Rha	10.2	10.0	9.9
Ara	57.5	47.6	54.8
Gal	24.7	33.7	26.7
Uronic acid	7.6	8.7	8.6

The data were shown as molar percentage. The presented numbers were average of two analyses.

Earlier work demonstrated that tomato pI 4.6 extensin peroxidase can catalyze *in vitro* cross-linking of P3 extensin analog YK_8_ and YK_20_ [17]. To test the cross-linking possibilities of 224, 242, and 442, each glycomodule was incubated with hydrogen peroxide and the pI 4.6 peroxidase isolated via DEAE-anion exchange chromatography as reported by Held et al. [17]. The reaction mixture was fractionated on an analytical Superose-6 size exclusion column to monitor the time-dependent decrease of 224, 242 and 442 monomers and the concomitant increase of multimers. The cross-linking rate was determined from calculating the loss of monomer during the reactions [17]. In the presence of 1 µg of peroxidase, the 224 and 242 glycomodules shared a cross-linking reaction rate of about 2,000 µg glycomodule/min, while that of 442 was nearly 4,000 µg/min, a rate comparable to that of the YK_8_ P3 extensin analog reported earlier. This result was consistent with our earlier results that larger extensin analogs cross-linked faster than smaller ones [17].

**Figure 2:**
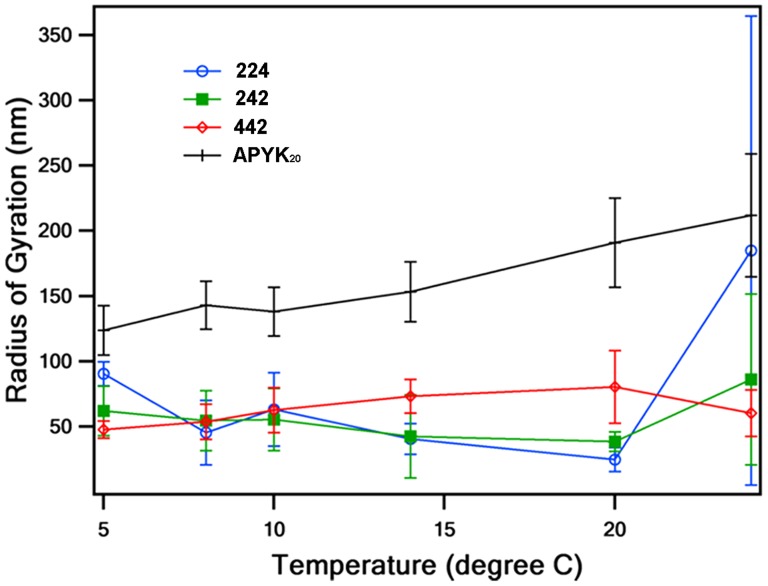
Temperature and molecular radius relationship of the glycomodules. When temperature increased from 20°C to 24°C, the average radii of 224 and 242 glycomodules increased as the control extensin analog APYK20. Only 442 glycomodule had a decreased average radius when the temperature increased from 20°C to 24°C. At 24°C, the standard deviation of 224 radius was greater than that of 242, with both standard deviations much greater than that of 442. It indicates that 224 and 242 glycomodules may be responding to temperature in a broader size range at 24°C.

Although elastins share an extended 3-dimensional structure, elastins fold to an ordered beta-spiral structure when the environmental temperature is higher than a certain point, the transition temperature [19]. If the fused elastin motifs in 224, 242, and 442 glycomodules would inherit this phase transition feature, the sizes of these glycomodules hence their possible stereo-structure in the walls would be temperature-dependent. We measured the radius of gyration of each glycomodule at different temperatures using dynamic light scattering [31]. When the temperature was increased from 20°C to 24°C, the average radii of 224 and 242 increased from 25 nm to 180 nm and from 40 nm to 80 nm, respectively, while the average size of 442 decreased to 60 nm (Fig. 2). The control extensin analog, APYK_20_ (hybrid glycoprotein (AlaHyp)_4_(SerHyp_4_SerHypSerHyp_4_TyrTyrTyrLys)_20_) [32], exhibited the same behavior as 224 and 242 after treatment. However, the standard deviations of the 224 and 242 radii measured at 24°C were much greater than that of the 442 radius, indicating that the 224 and 242 glycomodules may be responding to temperature in a broader size range at 24°C than at 20°C. Thus, only the 442 glycomodule might exhibit an elastin-like transition in response to temperature change.

### 2. Physical changes of the cell walls of transgenic tobacco cells

Cells expressing 224-EGFP and 242-EGFP yielded up to 15 mg of fusion glycoproteins per liter of culture media. In contrast, the *442-EGFP* transgenic tobacco cells produced 442-EGFP glycoprotein in a yield of more than 70 mg/L of culture media. Indeed, the culture medium of 20-day-old *442-EGFP* cells was dark green due to the accumulation of secreted EGFP fusion protein.

**Figure 3:**
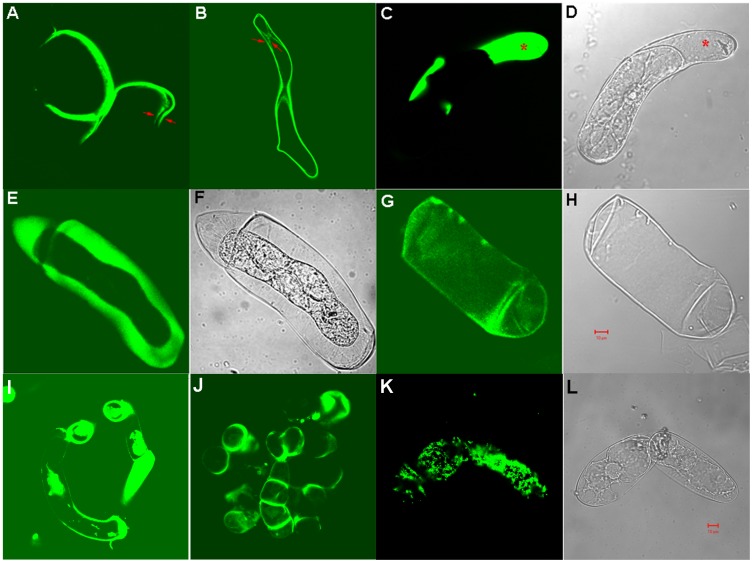
Transgenic cells under confocal microscope. Panels A to H were images of *442-EGFP* cells, among which cells in A to D were in liquid culture media. Arrows in A and B labeled the plasma membrane and cell wall respectively, showing 442-EGFP in the cell wall. The * labeled area of cell in C and D showed accumulation of 442-EGFP between the plasma membrane and the cell wall under normal suspension culture condition. Panels E and F were images of cells in 1 M mannitol, while G and H were cell walls prepared after sonication and wash with 2M NaCl and water. Panels I and J were images of *224-EGFP* and *242-EGFP* cells in 1 M mannitol, respectively; panels K and L were images of control *YL8-EGFP* cells in 1 M mannitol, in which the green fluorescence was mainly distributed on the plasma membrane. *YL8-EGFP* stands for gene encoding (SP_4_SPSP_4_YYYL)_8_-EGFP fusion protein [17]. Except panels D, F, H, and L were taken under normal light, other panels showed cells under excitation at 480 nm and emission at 520 nm. Scale bar: 10 µm. Panel A-H share the same scale bar, and panel I-L share the same scale bar.

The incorporation of EGFP fusion proteins into the cell walls was monitored by confocal microscopy and EGFP labeling of cell walls was evident (Fig. 3). It was also confirmed by the green fluorescence retained by the isolated *442-EGFP* cell walls (Fig. 3G and 3H). Furthermore, the high production of 442-EGFP led to partial separation of the plasma membrane from the walls of the *442-EGFP* cells when those cells were checked under normal culture conditions (Fig. 3C and 3D). After plasmolysis in 1M mannitol, the 442-EGFP fluorescence was distributed between the plasma membrane and the *442-EGFP* cell wall (Fig. 3E). The Hechtian threads were evident under plasmolyzing conditions (Fig. 3F) but absent under normal culture conditions (Fig. 3D), suggesting the green fluorescence distribution in Fig. 3C resulted from accumulation of the EGFP fusion protein in that area.

The deposition of the fusion proteins into the cell walls prompted us to further check the wall physical properties and chemical composition. One striking feature was the change of cell wall volume of *442-EGFP* cells, compared to that of wild type tobacco cells and walls isolated from the other transgenic lines, that arose when wall preparations were washed with NaCl solution and water (see methods). Specifically, after breaking 40 grams of filtered fresh cells by sonication, each preparation was centrifuged to pellet the crude walls. Initially, the packed cell wall volume (PCV) after centrifugation did not vary significantly between the screened cell lines (about 15 ml PCV for WT, 27 ml for *224-EGFP,* and 19 ml for *242-EGFP* cell walls), and nor were differences in PCV observed after washes with 1M NaCl (Table 3). However, the *442-EGFP* cell walls swelled dramatically in response to the water washes, the PCV increasing to 66 ml from 19 ml. Furthermore, the washed and pelleted *442-EGFP* walls were not ‘sticky’ as they could be poured easily, while the other walls after centrifugation formed pellets that were clumped and would not pour.

**Table 3:** Packed cell wall volume (PCV) of each cell line during cell wall preparation.

Sample	Wild Type Cells	*442-EGFP* Cells	*242-EGFP* Cells	*224-EGFP* Cells
Wet Weight (g)	40	40	40	40
PCV after sonication (ml)	15	22	19	27
PCV after 1M NaCl Wash (ml)	15	19	15	15
PCV after H_2_O Wash (ml)	15	66	23	25
Status after H_2_O Wash	Sticky	Not sticky	Sticky	Sticky
Dry Weight (mg)	234	672	464	446

### 3. Cell wall glycan changes

**Figure 4:**
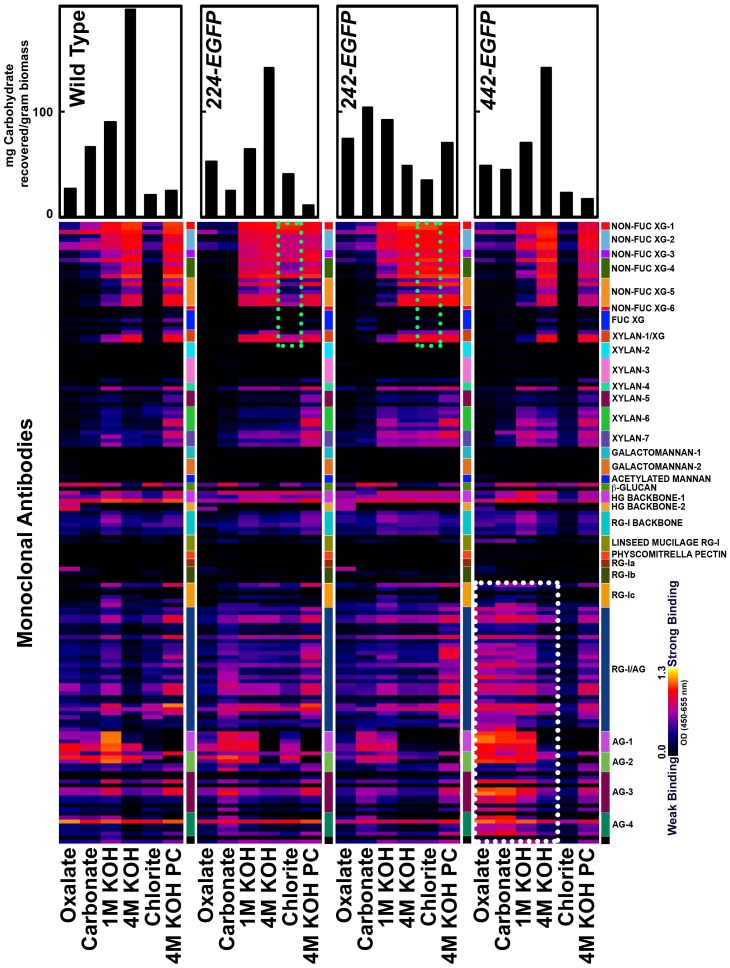
Glycome profiling of extracts from cell walls of transgenic tobacco cells versus wild type cells. Labels at the bottom show reagents used for the different extraction steps. The amounts of material extracted in each extraction step are indicated in the bar graphs at the tops of the heat maps. Extracts were ELISA-screened using 155 plant cell wall glycan-directed monoclonal antibodies [27], [28]. All ELISA wells were coated with 15 µl of a stock solution of each extract containing 20 µg/ml carbohydrate (glucose equivalents). Data are represented as heatmaps. The panel on the right of the heatmaps shows the antibodies that are grouped based on the principal cell wall glycans recognized [27], [28]. Strength of ELISA signal is indicated by black-blue-red-bright yellow scale with bright yellow depicting strongest binding and black indicating no binding. The results showed the early oxalate and carbonate extracts of both *442-EGFP* cell walls contained significantly increased arabinogalactan epitopes (dotted white box), in the AG1 to AG4 categories, and increased AG/RG-I epitopes (dotted white box). These data support that 442-EGFP fusion proteins were over-deposited into the walls. Also shown is the increased non-fucosylated xyloglucan epitopes in the 1 M KOH to post chlorite 4 M KOH extracts from *224-EGFP* and *242-EGFP* walls (dotted green boxes emphasized the chlorite fractions).

To further check the effect of extensin fusion protein deposition on the wall, glycome-profiling of cell wall extracts was used to compare the wall glycan epitopes between the transgenic versus wild type cells [27], [28]. Each cell wall preparation was extracted sequentially with 50 mM oxalate, 50 mM carbonate, 1M KOH, 4M KOH, acidic chlorite, and 4M KOH, which mainly extracts pectins (oxalate and carbonate), xyloglucans and xylans with varying amounts of strongly bound pectin (1 M and 4 M KOH), lignin associated glycans (chlorite), and remaining tightly bound wall glycans (post chlorite 4 M KOH). The amount of material (measured as dry weight) released from the cell walls of each cell line by each extractant varied significantly (Fig. 4, top bars), suggesting that the wall structures of all the transgenic lines were modified compared with WT walls. These extracts were ELISA-screened using 155 plant cell wall glycan-directed monoclonal antibodies. The results, presented in a heatmap, showed that the epitope compositions and extractabilities of xylan, glucan, homogalacturonan (HG) backbone, and rhamnogalacturonan-I (RG-I) backbone did not change significantly across all the samples. However, two obvious altered patterns were observed after comparing the heatmaps of the transgenic cell lines versus WT. The first altered pattern is that the oxalate and carbonate extracts from walls of the *442-EGFP* cells contained more arabinogalactan (AG) epitopes, including epitopes in the antibody groups AG1 to AG4 (dotted white box, Fig. 4) [27], [28], than WT, *224-EGFP*, and *242-EGFP* cell walls. The oxalate and carbonate extracts of *442-EGFP* cells also had increased AG/RG-I epitopes (dotted white box, Fig. 4). Because the total weight of the oxalate and carbonate extracts from *442-EGFP* walls was comparable to that of wild type, these data suggest that the extracts from *442-EGFP* cell walls contained more easily extractable arabinogalactan-containing polymers, consistent with the incorporation/deposition of 442-EGFP fusion proteins into the walls which were released, at least partially, from the walls with oxalate and carbonate extractions.

The second altered pattern is the dramatically increased presence of non-fucosylated xyloglucan epitopes in the chlorite extracts prepared from the *224-EGFP* or *242-EGFP* cell walls (dotted green box, Fig. 4) compared to the chlorite extracts from WT and *442-EGFP* walls. These data suggest that the degree of xyloglucan cross-linking in *224-EGFP* and *242-EGFP* cell walls was modified as a consequence of overexpression of the 224-EGFP and 242-EGFP extensin fusion proteins.

### 4. Chemical changes in the transgenic tobacco cell walls

The modification of cell walls through extensin fusion protein overexpression was further corroborated by analyses of Hyp and protein contents in the walls. Colorimetric Hyp assays showed that the *442-EGFP* cell walls contained significantly more Hyp (4.7 µg/mg of wall) than did the wild type walls (0.9 µg/mg of wall) (Table 4). The amount of Hyp in the *242-EGFP* walls also increased significantly. However that of the *224-EGFP* cell walls remained comparable to wild type walls (Table 4). Although amino acid composition analyses showed similar compositions for all walls analyzed (Table 5), the absolute amount of each amino acid was significantly higher in the *442-EGFP* walls with the total amino acid/protein content of *442-EGFP* walls accounting for 16.2% (w/w) of the dry wall weight, comparing to 2.9% (w/w) protein in wild type cell walls. This indicates that overexpression of 442-EGFP significantly increased the protein content of the wall, which might increase the hydration capacity of these cell walls and hence cause the PCV increase.

**Table 4:** Hydroxyproline, Tyr, Idt, and di-Idt amount in different cell walls.

Cell wall type	Hyp (µg)	di-Idt (nmol)	Idt (nmol)	Tyr (nmol)
Wild Type	0.91	1.92	2.19	5.11
*442-EGFP*	4.70	37.17	7.38	25.89
*242-EGFP*	2.93	3.62	2.37	7.57
*224-EGFP*	1.10	0.81	1.32	2.63

One mg of each sample was used for analysis. The presented numbers were average of two analyses.

**Table 5:** Amino acid composition of each cell wall (CW) preparation.

Amino acid Residue	Wild type CW		*442-EGFP* CW		*242-EGFP* CW		*224-EGFP* CW	
	nmol	mol%	nmol	mol%	nmol	mol%	nmol	mol%
D	1.05	9.8	6.47	10.8	1.57	10.0	0.41	9.2
E	1.01	9.4	6.14	10.2	1.40	8.9	0.32	7.1
O	0.69	6.4	2.10	3.5	0.99	6.3	0.43	9.6
S	0.84	7.8	4.47	7.4	1.20	7.7	0.34	7.6
G	0.79	7.3	5.08	8.4	1.15	7.3	0.32	7.1
H	0.21	2.0	1.13	1.9	0.29	1.9	0.08	1.8
R	0.34	3.2	2.42	4.0	0.53	3.4	0.11	2.5
T	0.43	1.0	3.11	5.2	0.65	4.2	0.17	3.8
A	0.89	8.3	5.41	9.0	1.31	8.4	0.32	7.1
P	0.78	7.3	3.80	6.3	1.20	7.7	0.34	7.6
Y	0.29	2.7	1.69	2.8	0.49	3.1	0.19	4.2
V	0.70	6.5	3.86	6.4	0.99	6.3	0.29	6.5
M	0.13	1.2	0.16	0.3	0.12	0.8	0.15	3.3
I	0.58	5.4	2.63	4.4	0.74	4.7	0.27	6.0
L	0.92	8.6	5.28	8.8	1.33	8.5	0.33	7.3
F	0.46	4.3	2.51	4.2	0.63	4.0	0.16	3.6
K	0.64	5.9	3.90	6.5	1.07	6.8	0.25	5.6
% (w/w) dry CW	2.9%		16.2%		4.3%		1.2%	

Each data set was collected from 40 µg of sample. O stands for hydroxyproline. The presented numbers were average of two analyses.

Pulcherosine and di-IDT are likely intermolecular cross-linking amino acids in extensins [14], [15]. The separation and quantification of Tyr and Tyr derivatives from the wall hydrolyzates showed a significant increase of di-IDT/IDT content in the *442-EGFP* walls. Wild type walls contained 1.92 nmol di-IDT/2.19 nmol IDT per mg of walls compared 37.17 nmol di-IDT/7.38 nmol IDT per mg of *442-EGFP* cell wall (Table 4). Interestingly, no significant amount of pulcherosine was detected in these samples. The *242-EGFP* cell walls also exhibited increased amounts of Tyr and Tyr derivatives (Table 4). However, the amount of Tyr derivatives in *224-EGFP* walls decreased with respect to wild type cell walls. These results suggest that the four tandem repeats of SerHyp_4_SerHypSerHyp_4_TyrTyrTyrLys P3 extensin motifs of 442-EGFP might allow more efficient cross-linking than 224-EGFP and 242-EGFP which each contain only two P3 extensin motifs in tandem (Fig. 1). These results are consistent with our speculation that a small portion of the 442-EGFP fusion glycoproteins that saturated the walls might have been cross-linked via the di-IDT cross-linking amino acids.

To further check the chemical basis of wall property changes, we analyzed the glycosyl residue compositions of the transgenic cell walls. Analyses of neutral sugars hydrolyzed with 2 N TFA and uronic acid residues hydrolyzed with concentrated H_2_SO_4_ showed that the *442-EGFP* cell walls contained similar amount of total monosaccharides as wild type (Table 6). Given the protein content in each wall preparation, we calculated that the total acid-hydrolysable carbohydrates account for 50.7% (w/w) of the non-protein portion of the *442-EGFP* walls, comparable to the 50.9% (w/w) for wild type walls. However, compared to wild type walls, the *442-EGFP* walls generally had less individual hydrolysable monosaccharides except for glucosyl residues, even after correction for the protein contents. Interestingly, the *224-EGFP* and *242-EGFP* walls contained significantly more glucosyl residues than wild type, with other monosaccharide compositions comparable to those of wild type and/or *442-EGFP* walls.

**Table 6:** Acid-hydrolysable sugar composition of cell walls.

Sugar residue	Wild type cell wall		*442-EGFP* cell wall		*242-EGFP* cell wall		*224-EGFP* cell wall	
	µg	nmol	µg	nmol	µg	nmol	µg	nmol
Rha	80.6	491	41.3	252	61.4	374	51.8	316
Fuc	17.3	105	3.0	18.0	0	0	0	0
Ara	140	933	99.2	661	155	1036	136	906
Xyl	66.5	443	50.1	334	61.9	413	59.2	395
Man	21.8	121	15.5	86.0	20.2	112	17.1	95.0
Glc	63.1	351	153	852	222	1236	300	1665
Gal	105	582	63.2	351	71.8	399	87.3	485
Uronic acid	274	1398	134	684	266	1357	197	1005
% (w/w)	49.4%		42.5%		59.2%		65.1%	

Each set of data was collected from 1 mg of sample. The presented numbers were average of two analyses.

**Figure 5:**
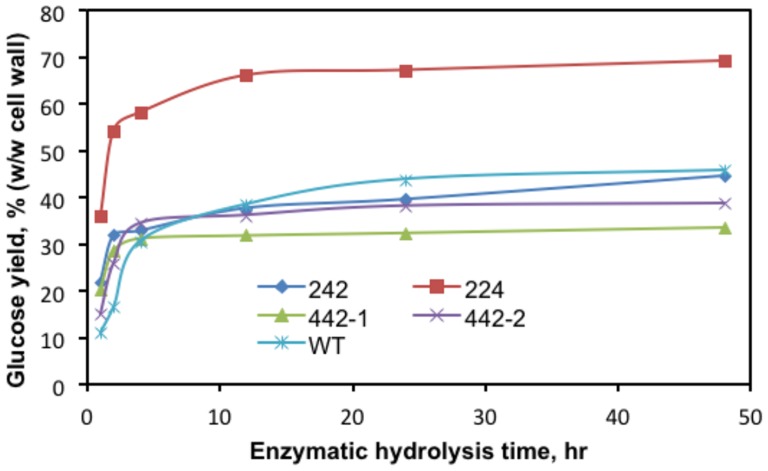
Sugar release of wild type versus *224-EGFP*, *242-EGFP*, and *442-EGFP* cell walls. Cell walls were digested with cellulase and glucosidase. The glucose contents in hydrolysis solution withdraw at time intervals of 1, 2, 4, 12, 24, and 48 hour were analyzed. The calibration of the system was performed with glucose standards. The *224-EGFP* cell walls showed a better sugar release performance than *242-EGFP*, *442-EGFP*, and wild type cell walls.

**Figure 6:**
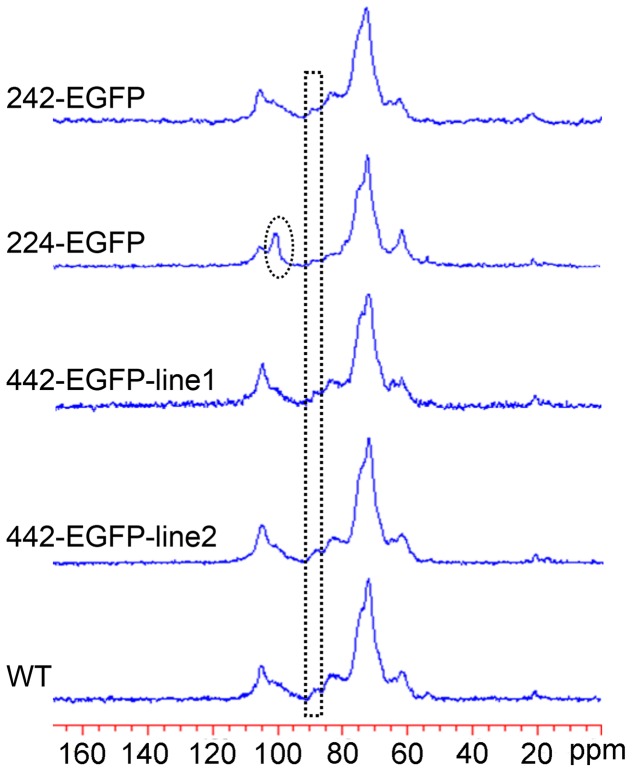
Solid state one dimensional ^13^C NMR spectra of walls of transgenic cells versus WT walls. The chemical shifts and relative signal intensities of anomeric carbons (i.e., C_1_) of glucosyl residues of cellulose in *242-EGFP* and *442-EGFP* cell walls were comparable to those of wild type cell walls. However, *224-EGFP* cell walls displayed much stronger signals of anomeric ^13^C of xylosyl residues (oval) and lower signal of crystalline cellulose (boxes).

Because TFA cannot hydrolyze crystalline cellulose, the different cell wall preparations were treated directly with cellulase and β-glucosidase to monitor the release of cellulosic glucose, which was quantified via HPLC cation exchange chromatography. Over the first five-hours of digestion, slightly more glucose was released from the walls of the *242-EGFP* cells and both *442-EGFP* cell lines than from WT walls (Fig. 5). When the digestions were extended to more than 12 hours, the cell walls of two *442-EGFP* lines and the *242-EGFP* cell walls yielded less glucose than the wild type walls (Fig. 5). However, the *224-EGFP* walls released much greater amounts of glucose than wild type walls at all incubation times, with a 70% (w/w) glucose yield after a one-day digestion. To further check if the crystallinity of each transgenic cell line was changed, one-dimensional ^13^C solid state NMR spectra were collected with the walls of transgenic cells versus WT (Fig. 6). The chemical shifts and relative signal intensities of anomeric carbons of cellulosic glucosyl residues in the *242-EGFP* and *442-EGFP* cell walls were comparable to those of wild type cell walls. However, the *224-EGFP* cell walls showed weaker C-4 signals of crystalline cellulose than the other walls (Fig. 6, boxes), suggesting lower cellulose crystallinity in the *224-EGFP* walls.

## Discussion

### Phenotype-chemotype changes of the three transgenic cell lines

Overexpression of the three extensin analogs in tobacco BY2 cells resulted in changes of primary cell wall properties. The wall phenotype-chemotype changes can be classified into three types based on the results of our physical and chemical analyses. One type of change was observed in the walls of *224-EGFP* cells, which exhibited decreased cellulose crystallinity (Fig. 6). The decreased cellulose crystallinity appears to be the primary factor leading to the reduced recalcitrance of the *224-EGFP* walls as measured by the significantly greater amounts of cellulosic glucose released from the transgenic walls after cellulase treatment compared with WT walls (Fig. 5). In addition, unlike the WT walls, the *224-EGFP* walls contained more xyloglucans in the chlorite wall extracts (Fig. 4), indicating that the expression of the 224-EGFP fusion protein led to cross-linking of xyloglucans to chlorite-susceptible polymers or substituents, which has never been observed in WT Arabidopsis walls. In addition, there appears to be an increase in the amount of xyloglucan in the walls of this transgenic cell line. The latter is consistent with both the solid state NMR data showing an increase of the Xyl anomeric carbon signal (Fig. 6, oval) and the sugar composition analysis documenting that the *224-EGFP* walls contained five-times more non-cellulosic glucose than WT (Table 6).

A second type of change was observed in the *242-EGFP* walls. Like the *224-EGFP* walls above, the *242-EGFP* walls contained chlorite-extractable non-fucosylated xyloglucan epitopes (Fig. 4) that are not found in chlorite extract of WT walls. However, the *242-EGFP* walls did not exhibit any changes in cellulose crystallinity (Fig. 6), nor did they exhibit reduced recalcitrance (as determined by cellulase-releasable glucose) (Fig. 5). Thus, the cross-linking of some xyloglucans to chlorite-susceptible wall components appears not to be a factor in recalcitrance.

A third type of wall change was exhibited by the *442-EGFP* cell walls. Walls from the *442-EGFP* cells showed a WT-like pattern of non-fucosylated xyloglucan epitopes in their glycome-profiles, but significantly more AG and AG/RG-I epitopes in the oxalate and carbonate extracts (Fig. 4). More dramatically, the *442-EGFP* cell lines secreted substantially more of the extensin fusion protein into the culture supernatant than did either of the other two transgenic cell lines. Furthermore, *442-EGFP* walls exhibited a dramatically increased packed cell wall volume (PCV) after water washes, which was not exhibited by either WT or the other transgenic cell walls. The *442-EGFP* walls also showed no reduced recalcitrance (Fig. 5), suggesting that the increased PCV phenotype did not affect any wall characteristics associated with recalcitrance.

### Possible mechanisms underlying the observed changes in phenotype-chemotype

The high production and secretion of the 442-EGFP fusion protein driven by the CaMV S35 promoter led to extensive accumulation of the EGFP-extensin fusion protein in the culture medium; the medium turned dark green after two-weeks of culturing. This result is consistent with our earlier reports that AGP glycosylation facilitates secretion of exogenous fusion proteins into culture media [33], [34]. We also observed the localization of green fluorescence between the plasma membranes and the cell walls of these cells (Fig. 3C and 3D), suggesting the overexpression of the *442-EGFP* gene construct results in saturation of the culture medium, the interspace between plasma membrane and cell wall, and the walls themselves with the 442-EGFP fusion protein. In addition, saturation of the walls with 442-EGFP, as reflected in higher (16%) protein content of the walls, might also saturate the extensin peroxidases that are responsible for cross-linking of functional endogenous extensins. The lack of endogenous wall extensin network consequently resulted in loosen cell walls, contributing to the increased PCV or hydration capacity of the *442-EGFP* walls.

In contrast, overexpression of the *224-EGFP* and *242-EGFP* gene constructs resulted in much lower accumulation of the corresponding fusion proteins in the culture media than was observed for the *442-EGFP* cells. We did not observe EGFP fluorescence saturated *224-EGFP* and *242-EGFP* cells as we documented in Fig. 3C. In addition, the 224 and 242 modules are less cross-linkable based on cross-linking rates measured *in vitro* for 224, 242, and 442, suggesting that 224-EGFP and 242-EGFP might not be ideal substrates for the extensin peroxidases thought to be responsible for cross-linking extensins in the wall. Thus, the endogenous extensins in these two transgenic cell lines could form regular extensin network for wall integrity, which might be why the *224-EGFP* and *242-EGFP* cells showed similar PCV as WT cells.

Furthermore, although the 224 and 442 glycomodules shared the same molecular mass and the same total content of each motif, including 8 YK cross-linking motifs, 4 AOAOAOA AGP motifs, and 16 VPGVP elastin motifs, the faster *in vitro* cross-linking rate of 442 suggests the two (YK)_4_ units in 442 were more cross-linkable than the four (YK)_2_ units in 224. This might be a reason that the *442-EGFP* cell walls contained more di-IDT content than WT or the other transgenic cell lines. However, why did the *242-EGFP* cell walls contain more di-IDT than the *224-EGFP* cell walls as 242 had only two (YK)_2_ units? One possible reason is that the more clustered AGs on 242 glycomodules might be a favorite factor for *in vivo* cross-linking given the adhesion property of AGs favoring intermolecular adhesion and alignment [35]. Moreover, why did overexpression of 224-EGFP in the *224-EGFP* cells result in less di-IDT content than WT? The elastin motifs in 224-EGFP might play a central role in this case, most likely due to the observed wide-range size change of the 224 glycomodule at 24°C attributed to the stretch-fold conformations of the 16 VPGVG motifs in each 224-EGFP. The conformations of elastin motifs in each extensin fusion protein might introduce some steric space into the walls, which would explain why all the transgenic cell walls released more cellulosic glucose than WT during the first five-hour cellulase digestion, as the cellulases could more easily access cellulose microfibrils near the steric spaces of those transgenic walls (Fig. 5). Overall, the total effect of each extensin fusion protein on its transgenic cell walls was a collective effect of all the three motifs.

Besides the change in hydration properties, the biomass of *442-EGFP* walls increased to thrice that of wild type walls (Table 3). This may result from either the deposition of 442-EGFP fusion glycoproteins, or increased endogenous wall polysaccharides in the walls, or both. The significantly higher Hyp and Tyr contents in the *442-EGFP* walls (Table 4), as well as greater than five-fold increase in protein content compared to that of wild type walls is consistent with the biomass increase being due, at least partially, to the over-deposition of 442-EGFP. However, we also observed that the biomass of *224-EGFP* and *242-EGFP* cell walls almost doubled compared to that of wild type cell walls (Table 3). Although the Hyp and protein contents of *242-EGFP* walls were slightly higher than that of wild type, the amounts of Hyp and protein in *224-EGFP* walls were even less than those of wild type. Therefore, the biomass increase in these two cases might mainly result from the increase of endogenous wall polysaccharides.

Finally, identifying peptide fragments of the extensin analogs from the walls of transgenic cells would provide direct evidence for their wall deposition. However, LC-MS/MS analysis of the total tryptic peptides generated from hydrogen fluoride (HF)-deglycosylated *442-EGFP* cell walls did not yield any peptide sequence attributable to 442-EGFP. It may be due to either the cross-linking of 442-EGFP in the walls (evident in high content of di-IDT) or the large size of the tryptic 442 peptides (Fig. 1). Nevertheless, labeling of the walls with green fluorescence, changes of amounts of wall di-IDT and protein, and changes of wall glycans in the walls of transgenic cells are consistent with deposition/incorporation of extensin fusion proteins into the walls.

## Conclusion

Overexpression of extensin analogs in tobacco primary cell walls resulted in changes of wall properties that were distinct for each transgenic cell line. The arrangement and sizes of the extensin motifs, AGP motifs, and elastin motifs on the polypeptide affected the effects on overall wall structure for each line. The *442-EGFP* walls showed no change in wall cellulose crystallinity; however, wall saturated with 442-EGFP may have prevented the formation of endogenous extensin network, which significantly changed wall physical and chemical properties. In contrast, the *224-EGFP* walls exhibited a higher ratio of xyloglucans to celluloses and lower cellulose crystallinity, most likely due to the spatial alterations caused by deposition of the more extended 224-EGFP fusion glycoproteins. However, the *242-EGFP* walls only displayed changes in xyloglucan extractability. These results indicate that regulated expression of modified extensins in *planta* may be a way to manipulate wall architecture to achieve higher biomass and higher biofuel production.
